# Polypropylene Crystallinity Reduction through the Synergistic Effects of Cellulose and Silica Formed via Sol–Gel Synthesis

**DOI:** 10.3390/polym16202855

**Published:** 2024-10-10

**Authors:** Gulbarshin K. Shambilova, Rinat M. Iskakov, Aigul S. Bukanova, Fazilat B. Kairliyeva, Altynay S. Kalauova, Mikhail S. Kuzin, Egor M. Novikov, Pavel S. Gerasimenko, Igor S. Makarov, Ivan Yu. Skvortsov

**Affiliations:** 1 Institute of Petrochemical Engineering and Ecology named after N.K, Atyrau Oil and Gas University named after S, Atyrau 060027, Kazakhstan; shambilova_gulba@mail.ru (G.K.S.); r.iskakov@aogu.edu.kz (R.M.I.); bukanova66@mail.ru (A.S.B.); kairlieva.fazi@mail.ru (F.B.K.); skalauova@mail.ru (A.S.K.); 2 Department of Chemistry and Chemical Technology, Kh. Dosmukhamedov Atyrau University, Atyrau 060011, Kazakhstan; 3 Department of Chemical and Biochemical Engineering, Satbayev University, Satbayev Street, 22, Almaty 050013, Kazakhstan; 4 A.V. Topchiev Institute of Petrochemical Synthesis, Russian Academy of Sciences, 119991 Moscow, Russia; kuzms@ips.ac.ru (M.S.K.); gerasimenko11507@yandex.ru (P.S.G.); makarov@ips.ac.ru (I.S.M.); 5 Department of Chemistry, New Mexico Highlands University, Las Vegas, NM 87701, USA; enovikov@live.nmhu.edu

**Keywords:** cellulose, oligosiloxanes, polypropylene, composites, rheology, dynamic thermomechanical analysis, polycondensation, crystallinity, sol–gel synthesis

## Abstract

This study focuses on the development of environmentally sustainable polypropylene (PP)-based composites with the potential for biodegradability by incorporating cellulose and the oligomeric siloxane ES-40. Targeting industrial applications such as fused deposition modeling (FDM) 3D printing, ES-40 was employed as a precursor for the in situ formation of silica particles via hydrolytic polycondensation (HPC). Two HPC approaches were investigated: a preliminary reaction in a mixture of cellulose, ethanol, and water, and a direct reaction within the molten PP matrix. The composites were thoroughly characterized using rotational rheometry, optical microscopy, differential scanning calorimetry, and dynamic mechanical analysis. Both methods resulted in composites with markedly reduced crystallinity and shrinkage compared to neat PP, with the lowest shrinkage observed in blends prepared directly in the extruder. The inclusion of cellulose not only enhances the environmental profile of these composites but also paves the way for the development of PP materials with improved biodegradability, highlighting the potential of this technique for fabricating more amorphous composites from crystalline or semi-crystalline polymers for enhancing the quality and dimensional stability of FDM-printed materials.

## 1. Introduction

Polymer composite materials are highly valued for their exceptional combination of strength, thermal insulation properties, low density, and cost-effectiveness, making them indispensable across various modern applications [1,2,3]. Composite materials have existed for centuries [4], and their fundamental principles remain unchanged: they consist of fillers (fibers, minerals, metals, etc.) embedded within a matrix, which is often a thermoplastic or thermosetting polymer, along with various modifiers and additives [5].

Polypropylene (PP) is a popular choice as a composite matrix due to its high chemical resistance, mechanical strength, and low cost, which is further enhanced by its recyclability [6]. Numerous studies have explored the incorporation of fibers [7,8,9,10] and solid particles [11,12,13,14,15] into the polypropylene matrix to improve mechanical [9,11,16] and thermal insulation [12,13,17], and other [14,15,16,18] properties of the resulting materials. One of the key areas of research is the modification of polypropylene to enhance its biodegradability [19,20,21,22].

One effective method to increase the biodegradability of PP is the incorporation of cellulose fibers into its melt [23,24]. Cellulose is a renewable and cost-effective source of fibers [25,26,27]. Its properties, such as the degree of polymerization, lignin content, crystallinity, and hygroscopicity, vary depending on the source and processing method, which can significantly influence the properties of the composite materials [16,28,29]. The most commonly used cellulose fibers in composites are those produced by the Lyocell [29,30,31,32] and viscose [33,34,35] processes, as well as carbonized fibers [8,36,37], which are known for their high modulus of elasticity and strength [38,39,40].

A major challenge in composite materials is interfacial adhesion. In the case of a polypropylene matrix with cellulose filler, adhesion can be enhanced through various additives. For instance, it has been demonstrated that adding maleic anhydride-modified polypropylene significantly improves interfacial adhesion and simplifies the dispersion of cellulose in the polypropylene matrix, allowing for up to 90 wt.% cellulose content [28]. Other studies have shown that adhesion can be further improved by modifying cellulose through various methods. An intriguing alternative for enhancing interfacial adhesion is the addition of small, rigid particles. For example, submicron SiO_2_ spheres have been shown to significantly improve the interfacial adhesion between cellulose and polypropylene [41,42].

However, achieving a uniform distribution of components with varying stiffness and size in a highly viscous solution is a challenging task [43]. This challenge can be mitigated by sol–gel synthesis of submicron SiO_2_ particles via polycondensation of organosilicon compounds [44,45,46,47]. This technique has proven effective in the solution-based fabrication of SiO_2_-filled fibers [48]. Notably, the addition of organosilicon compounds often reduces the viscosity and elasticity [43], thereby simplifying the mixing and homogenization of complex systems. This sol–gel synthesis of SiO_2_ has been used to produce composites filled with SiO_2_ and reinforced with cellulose [44]. In another study, SiO_2_ particles were synthesized directly on cellulose nanofibers, which were then incorporated into ultra-high-molecular-weight polyethylene, leading to significant improvements in the mechanical and frictional properties of the resulting materials [42]. Despite these advances, the synthesis of such composite materials remains highly complex, involving multiple stages and solvent exchange. Furthermore, the use of solid particles can complicate processing due to the increased viscosity and elasticity of the melt.

PP is a partially crystalline polymer; therefore, during the cooling of the melt, it is prone to uneven shrinkage due to crystallization. This leads to significant issues, especially when printing large products using 3D printing. One potential solution to improve print quality could be the polymer crystallinity decreasing.

The objective of this study is to develop a formulation and a simple one-step method for producing advanced composite materials based on silica-filled thermoplastic reinforced with cellulose for use in fused deposition modeling (FDM) 3D printing with polypropylene. The inclusion of cellulose in the composite enhances mechanical properties and biodegradability, while silica nanoparticles improve adhesion, facilitate the mixing process, and enhance the overall performance. The uniqueness of the approach lies in the use of oriented solid microfibers, which serve as a reinforcing phase on one hand and as a source of a small amount of water for the hydrolytic polycondensation of alkoxysilane on the other. This approach has enabled the hydrolytic polycondensation to occur directly in the melt of hydrophobic polypropylene, which has not been previously described in the literature. This approach reduces the crystallinity and shrinkage of the composites, offering a viable alternative to polylactide plastics and improving the biodegradability of complex polypropylene-based products.

## 2. Materials and Methods

### 2.1. Materials

Polypropylene (trademark H030GP, Sibur, Russia) was used as the matrix material. Viscose fibers (Aditya Birla Group, Mumbai, India) were employed as the reinforcing fibers, containing 8 wt.% moisture, as determined using thermogravimetric analysis MS-70 (A&D, Tokyo, Japan). Olygosiloxane (ethyl silicate 40 (ES-40), Silane, Moscow, Russia) was introduced into the composite materials as a silica precursor. The polycondensation of ES-40 was carried out by adding a mixture of ethanol 98% (ECOS-1, Moscow, Russia) and distilled water in a 4:1 mass ratio.

### 2.2. Methods

#### 2.2.1. Preparation of Blends

In the initial stage, a mixture of cellulose and ES-40 was prepared in the required ratio (Table 1), and the first column denotes the sample name, which characterizes the ratio of cellulose to ET-40. In the total mass of the sample, cellulose and ET-40 comprise 15%. For some of the samples, a water–ethanol mixture in a 2:1 ratio relative to ES-40 was added (samples with ethanol addition are marked with the index “E” in Table 1) and maintained for 4 min at 60 °C. Subsequently, granulated polypropylene was added to the mixture, making up 85 wt.% of the resulting composite. The obtained mixture was blended in a laboratory twin-screw extruder (Haake Minilab 3, Thermo Scientific, Karlsruhe, Germany) under recirculation mode for 10 min at 220 °C.

#### 2.2.2. Rheological Analysis

The rheological properties of the prepared samples were studied using a MARS-60 rotational rheometer (Thermo Scientific, Karlsruhe, Germany) with a plate−plate geometry, featuring a 20 mm diameter plate (gap set at 1 mm). For all samples, viscoelastic properties were measured across strain amplitudes from 0.001% to 10% at a frequency of 1 Hz. In the linear viscoelastic region, frequency sweeps of storage modulus (G’) and loss modulus (G’’) were performed over a range of 628 to 0.63 rad∙s^−1^.

The dimensional stability of the samples was assessed using a rotational rheometer, under a constant normal force, which was determined in a preliminary experiment to maintain a constant gap at 200 °C. This experiment was conducted during cooling from 200 °C to 90 °C at a rate of 5 K∙min^−1^.

#### 2.2.3. Optical Microscopy

The morphology of the composite materials was examined on thin films with a thickness of 10 μm. Images were captured using a “Biomed 6PO” microscope (“Biomed”, Moscow, Russia), equipped with a ToupTek E3ISPM5000 camera (“ToupTek Photonics Co.”, Hangzhou, China) and a Plan-Apo objective with 4x magnification in both bright-field and cross-polarized light.

#### 2.2.4. Differential Scanning Calorimetry (DSC)

The thermal behavior of the samples was studied using a DSC 2920 Modulated Differential Scanning Calorimeter (TA Instruments, New Castle, DE, USA). The initial heating stage involved raising the temperature from 20 °C to 220 °C at a rate of 10 K·min^–1^, followed by cooling back to the starting temperature at 20 K·min^–1^. A second heating cycle was performed up to 220 °C at 10 K·min^−1^ to investigate crystallization under uniform conditions.

## 3. Results and Discussion

### 3.1. Rheology

**Figure 1:**
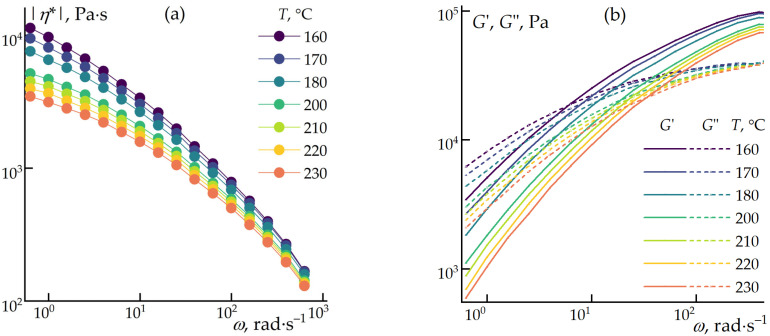
Dependencies of complex viscosity (|η^*^|) (**a**) and storage (*G*′), and loss (*G*″) moduli (**b**) on angular frequency at 0.1% strain.

In the initial phase of this study, the optimal blending temperature for the composites was determined by measuring the viscoelastic properties of polypropylene across a temperature range of 160 to 230 °C (Figure 1). The goal was to identify the lowest possible temperature that would minimize the evaporation of ES-40 while avoiding thermal degradation of the cellulose during mixing.

It can be observed that the polypropylene sample begins to flow at 160 °C, and, by 200 °C, its viscosity decreases to 5000 Pa·s. This reduction in viscosity should promote adequate wetting of the filler particles and create favorable conditions for processing in the extruder. The crossover point, where the storage and loss moduli are equal, occurs at approximately 10 rad·s^–1^, indicating a broad frequency (and strain rate) range where the polymer exhibits viscoelastic behavior [49]. This is crucial for achieving homogeneous compositions during mixing.

**Figure 2:**
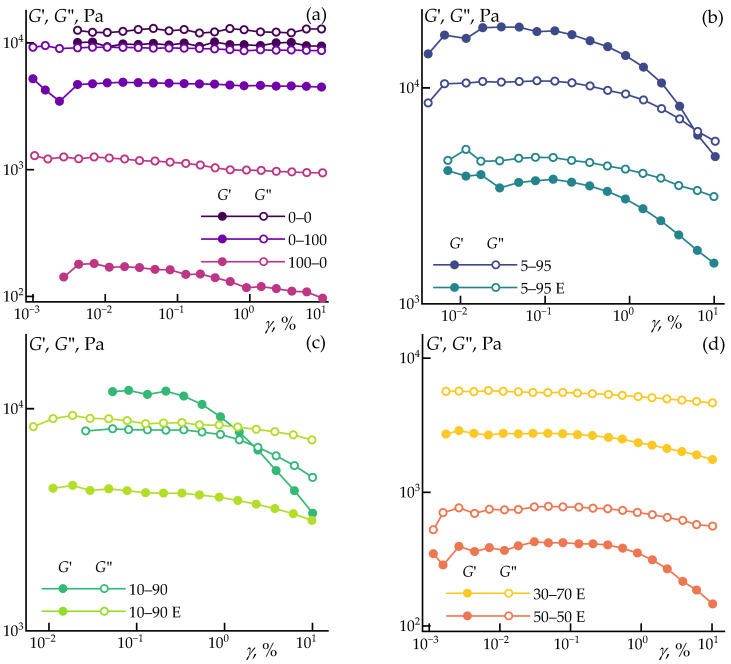
The amplitude dependencies of the storage and loss moduli of the composites at the selected processing temperature of 200 °C. (**a**) comparison of neat PP, PP with cellulose and PP with ES-40, (**b**,**c**) comparison of systems with preliminary HPC (samples with E) and HPC in the melt for 5–95 and 10–90, respectively, (**d**) comparison of 30–70 E and 50–50 E compositions.

The amplitude dependencies of the storage and loss moduli of the composites are shown in Figure 2.

The neat polypropylene (sample 0–0), polypropylene with 15% ES-40 (sample 0–100), and polypropylene with cellulose (sample 100–0), as well as the blends 10–90 E and 30–70 E, exhibit the widest linear viscoelastic region. This indicates a strong resistance to deformation without failure, which is a desirable trait for these samples. The linear viscoelastic range of samples 5–95 E and 50–50 E is limited to 0.3% and 1% strain, respectively. For all the aforementioned samples, the loss modulus exceeds the storage modulus, indicating that these samples exhibit flow behavior under small deformations.

Samples 5–95 and 10–90 are of particular interest, as they exhibit the characteristics of viscoelastic solids. Within the linear viscoelastic region, these samples have a storage modulus higher than the loss modulus and exhibit yield and flow points of 0.3% and 9% and 0.3% and 2%, respectively. This means that these samples behave as solids under small deformations and begin to flow when a certain shear is applied [50], making them suitable for manufacturing products through injection molding or extrusion from such structured systems.

**Figure 3:**
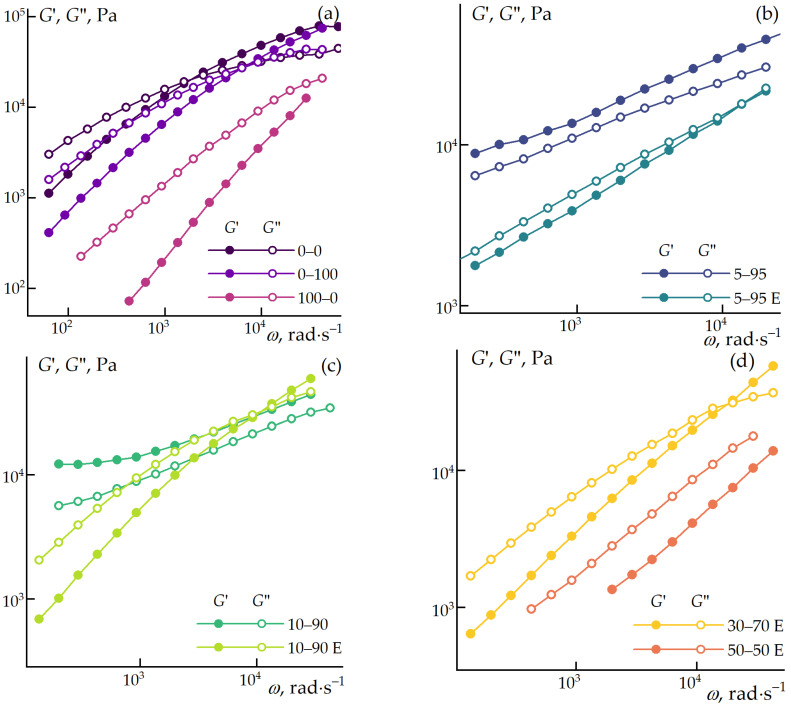
Frequency dependence of storage and loss moduli for blended composites at 200 °C (0.1% strain). (**a**) comparison of neat PP, PP with cellulose and PP with ES-40, (**b**,**c**) comparison of systems with preliminary HPC (samples with E) and HPC in the melt for 5–95 and 10–90, respectively, (**d**) comparison of 30–70 E and 50–50 E compositions.

Based on the conducted experiments, a strain of 0.1% was selected for all samples to measure the frequency dependence of the moduli. The results are presented in Figure 3.

At 200 °C, the neat polypropylene exhibits viscoelastic liquid behavior with characteristic Maxwellian behavior—slopes of 2 and 1 for the storage and loss moduli, respectively, in the terminal zone, and a crossover point at 20 rad∙s^−1^, above which the sample predominantly exhibits elastic behavior, and such viscoelastic behavior are preferable for fiber spinning. The introduction of 15% ES-40 leads to an equal reduction in both the storage and loss moduli of the composite and shifts the crossover point to higher frequencies (up to 100 rad·s^–1^). A more pronounced reduction in the components of the complex modulus is observed with the addition of 15% solid cellulose. The moduli values decrease tenfold compared to the neat polypropylene, and the crossover point shifts to even higher frequencies, reaching approximately 300 rad·s^−1^. This behavior is likely due to interfacial slippage between the polymer and cellulose particles or the formation of polyoligosiloxane particles, which are formed from ES-40, presumably due to incomplete hydrolysis in the absence of sufficient water in the hydrophobic polypropylene matrix.

The simultaneous introduction of ES-40 and cellulose significantly affects the viscoelastic properties of the composites. The sequence of additive incorporation determines the rheological properties of the blend. The simultaneous addition of ES-40 and cellulose into the extruder likely promotes the formation of a large number of particles due to hydrolysis and condensation in the viscous melt. In this case, the water required for hydrolysis is provided by the cellulose (10%), and the growth of particles is limited by low diffusion rates. The resulting particles, similar to silica additives, contribute to thickening the composite and forming structured systems (samples 5–95 and 10–90). In systems where the sol–gel reaction has been partially carried out in the presence of ethanol and water, the particles are formed in a liquid medium, which promotes their growth and results in larger interparticle distances in the composite. This effect is associated with the hydrolytic polycondensation of ES-40, resulting in the formation of SiO_2_ in the presence of water or ethanol [51,52]. Therefore, during hydrolysis in an excess of water or alcohol (liquid phase), larger particles are formed. It is known that as particle size increases, their specific surface area decreases, leading to a reduction in the interfacial boundary and an increase in the distance between particles [53,54,55]. This is also indirectly confirmed rheologically (Figure 3). In the case of larger interfacial distances in the composite, the mobility of individual structural elements increases, which in turn reduces the elasticity of the solution, bringing the system closer to a Maxwell viscoelastic liquid state. Conversely, when the distances are smaller, the mobility of structural elements decreases, the system’s elasticity increases, and it exhibits viscoelastic (gel-like) behavior. In these systems, the influence of silica and cellulose is significantly less pronounced, and they exhibit behavior typical of weakly structured systems with storage and loss moduli considerably lower than 2 and 1, respectively, in the terminal zone. The degree of structuring is most apparent in sample 5–95 E, where the concentration of ES-40 is the highest.

### 3.2. Morphology

**Figure 4:**
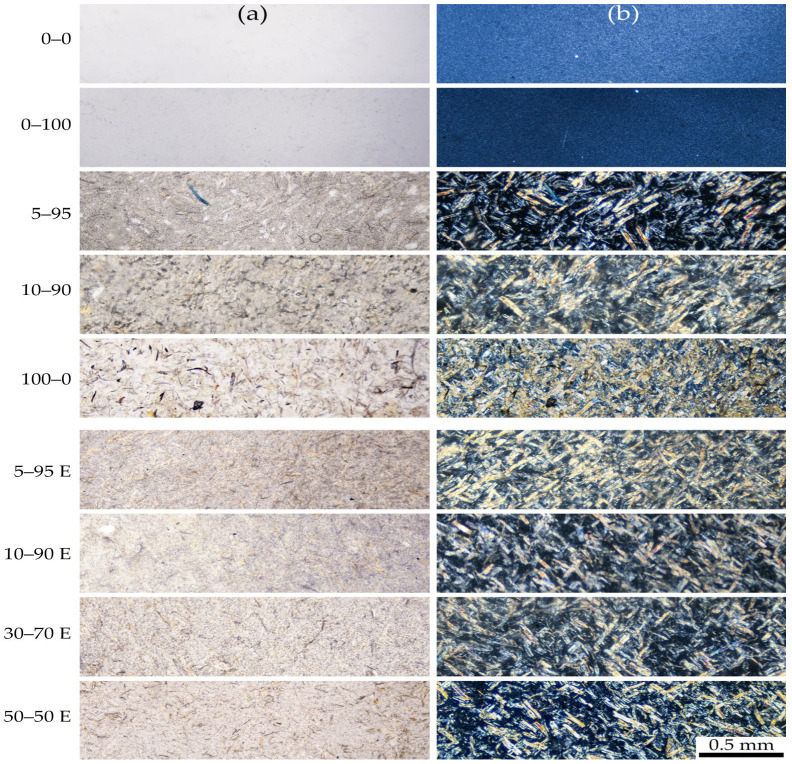
Optical microscopy of composite films captured in bright field (**a**) and crossed polarizers (**b**).

Figure 4 presents micrographs of composite films with uniform thickness (100 µm) captured in both bright field and crossed polarizers.

The micrographs reveal that polypropylene is a partially crystalline polymer, as evidenced by its uniform brightness under crossed polarizers. The addition of ES-40 to the blend did not lead to the appearance of any particles visible under the optical microscope. Under crossed polarizers, a decrease in the intensity of birefringence was noted for films of the same thickness and under identical imaging conditions. This suggests a reduction in the crystallinity of polypropylene in the presence of particles formed as a result of ES-40 hydrolysis.

The introduction of cellulose significantly alters the morphology of the composites, as expected. In all cases, cellulose appears to be uniformly distributed throughout the blend, which is clearly visible from the intensely birefringent cellulose fibers under crossed polarizers. Despite following a uniform mixing protocol, blends prepared with ethanol and water, which may cause slight swelling of cellulose, exhibit a more homogeneous fiber distribution. It is likely that as cellulose fibers swell, their stiffness decreases, making them more susceptible to deformational effects, which facilitates mixing.

The experiment confirms the feasibility of directly mixing the PP-cellulose-ES-40 compositions in the extruder.

### 3.3. Differential Scanning Calorimetry (DSC)

**Figure 5:**
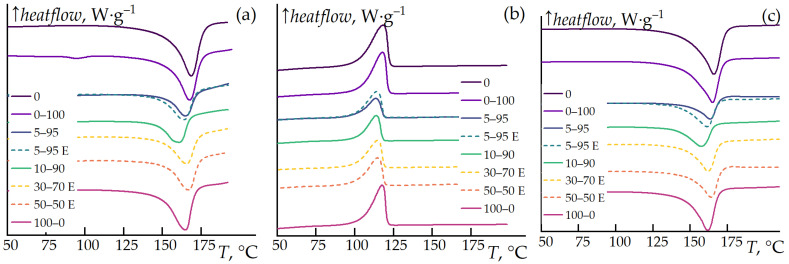
DSC curves of neat PP, as well as its blends with cellulose and ES-40. First heating (**a**), cooling (**b**), and reheating (**c**) at a rate of 10 K∙min^−1^. Samples with pre-hydrolysis before mixing with PP are marked with dashed lines.

The effects of the additives on the crystallization and melting processes of the composite mixtures were investigated. The experiments were conducted in the first heating, cooling, and reheating modes to eliminate the influence of crystallization conditions during sample preparation. The results are presented in Figure 5.

The thermal effects observed during the first and second heating cycles are identical, characterized by a distinct endothermic peak associated with the melting of PP. The addition of 15% cellulose or 15% ES-40 to PP has a weak effect on the crystallization process. However, the combined addition of ES-40 and cellulose results in significant changes in both the crystallization/melting temperatures and a pronounced reduction in the crystallization enthalpy of PP.

The behavior of the 5–95 and 5–95 E samples differs only slightly; the pre-hydrolyzed sample exhibits a slightly higher thermal effect.

**Figure 6:**
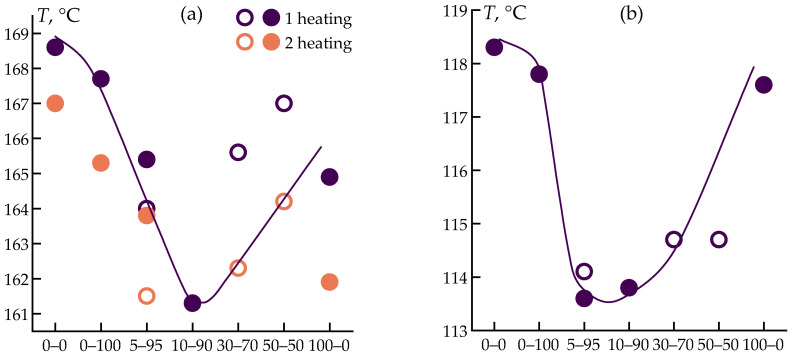

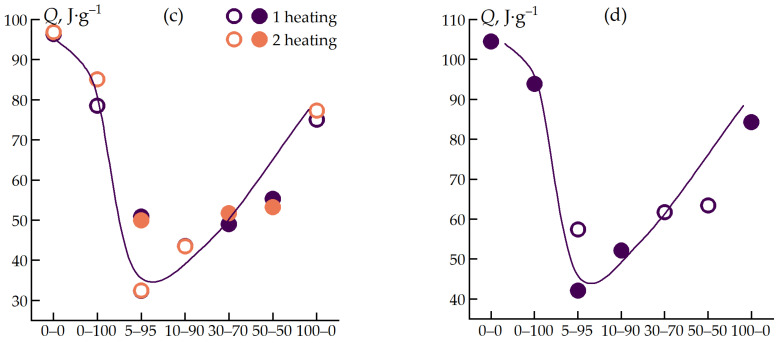
Changes in temperature and thermal effects during heating (**a**,**c**) and cooling (**b**,**d**) for the samples under study. The horizontal axis shows the names of the samples.

Based on the experiments, the integrated thermal effects of the peaks (enthalpy) and the peak temperatures for melting and crystallization were determined. The data are presented in Figure 6.

Polypropylene exhibits a distinct melting endotherm at 168 °C and a crystallization exotherm at 118 °C, with a thermal effect of approximately 100 J·g^−1^. The addition of cellulose or ES-40 to PP has minimal impact on the melting or crystallization temperatures and the thermal effect. However, the simultaneous addition of ES-40 and cellulose reduces the melting temperature of the composite by up to 7 degrees and the crystallization temperature by up to 4 degrees. The most significant impact is observed in the synergistic effect of introducing both ES-40 and cellulose, which drastically reduces the crystallization thermal effect of PP. For the 5–95 blend, the thermal effect decreases from 100 J·g^−1^ to 40 J·g^−1^, indicating a significantly lower degree of crystallinity in the resulting composite.

The energy required for melting and crystallization of the samples is consistent, indicating that the experiments were conducted correctly, with no side processes occurring within this temperature range.

### 3.4. Dynamic Thermomechanical Analysis (DTMA)

Uniform shrinkage during cooling is critical to avoid the formation of irregularities or the buildup of stresses during FDM 3D printing. Therefore, printing with crystallizing polymers is challenging and often requires specialized equipment [56,57,58].

**Figure 7:**
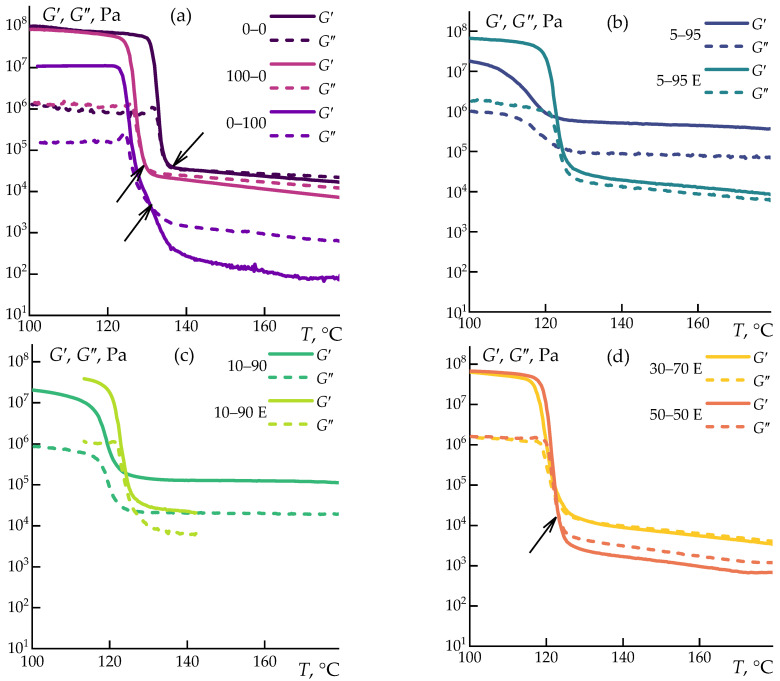
Temperature dependence of storage and loss moduli (**a**) 0–0/100–0/0–100; (**b**) 5–95/5–95 E; (**c**) 10–90/10–90 E; (**d**) 30–70 E/50–50 E. The example of crossover temperature is indicated by arrows in (**a**,**d**).

To study the thermal expansion behavior of the composites alongside changes in viscoelastic properties during cooling, the mixtures were examined using a rotational rheometer, which served as a precise thickness gauge under minimal load. The experiment was combined with oscillation (1 Hz, 0.1% strain) to accurately determine the crystallization temperature by monitoring the increase in storage and loss moduli (data presented in Figure 7).

The experiments indicate differences in the crystallization kinetics of the samples. Neat PP crystallizes rapidly, within a temperature range of less than 5 degrees from the onset to the completion of the process. The addition of 15% cellulose broadens this range to 10 degrees, with the storage modulus starting to increase at the same temperature but completing the process later. Adding 15% ES-40 shifts the onset of crystallization to lower temperatures, but the temperature range remains narrow, around 5 degrees. This minimal effect is likely due to the limited extent of hydrolysis under water-deficient conditions in neat PP. Similar behavior is observed in all pre-hydrolyzed solutions in the alcohol–water mixture (E series). For these, crystallization begins at 126 °C and ends at 116 °C. The most significant change in solidification rate is observed in the 5–95 and 10–95 samples, characterized by a gradual increase in moduli over a wide temperature range.

**Figure 8:**
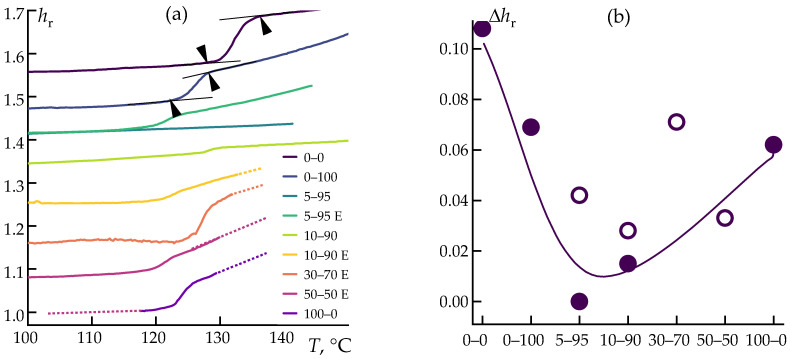
Temperature dependence of relative sample thickness (**a**), and thickness deviation for the composites under study (**b**). The horizontal axis shows the names of the samples.

During cooling under constant normal force, the change in sample thickness was measured. The most interesting region, within the solidification range, is shown in Figure 8. Here, *h*_r_ = *h*_i_/*h*_0_, Δ*h*_r_ = *h*_rmax_ − *h*_rmin_, where *h*_i_ is the current thickness, *h*_0_ is the initial film thickness, and *h*_rmax_ and *h*_rmin_ are the film thicknesses within the sample’s solidification range (an example is marked with arrows in Figure 8).

Crystallization leads to a sharp change in the geometric dimensions of the samples. However, the addition of pure cellulose or pure ES-40 to the composition does not significantly alter the shrinkage values. The simultaneous presence of both ES-40 and cellulose in the blend significantly reduces shrinkage (by more than twofold), with this effect being most pronounced in the 5–95 and 10–90 samples. This is likely due to the formation of a large number of silica nanoparticles from the hydrolysis of ES-40 within the PP matrix, resulting in a complex structural network that hinders PP crystallization. This network effectively minimizes the non-uniform shrinkage of the composite blend to nearly negligible levels.

The substantial differences in the behavior of samples obtained through pre-hydrolysis versus in situ sol–gel synthesis within the extruder can be attributed to kinetic factors. ES-40, similar to its oligomer tetraetoxysilane, undergoes active hydrolysis due to the residual moisture present in cellulose, with this process being accelerated at elevated temperatures. At the temperature used for the preparation of these compositions, the expected time for hydrolytic polycondensation is approximately 10 min [59]. As a result, silica particles form within the polymer matrix [48]. In the case of pre-hydrolysis, the low viscosity of the medium (an ethanol–water solution with cellulose particles) and the high diffusion rates of the components facilitate the coalescence of hydrolyzed ES-40 droplets into relatively large sizes, followed by the formation of larger particles upon condensation. These larger particles have a smaller interfacial surface area, which reduces their impact on the behavior of the composite blends.

## 4. Conclusions

The behavior of composite blends based on polypropylene (PP) with the addition of the oligomeric siloxane precursor ES-40 and cellulose was investigated using methods such as rotational rheometry, optical microscopy, differential scanning calorimetry, and dynamic mechanical analysis. It was demonstrated that hydrolytic polycondensation plays a pivotal role in altering the structural and thermal properties of PP composites. The results show that both methods of polycondensation—either via a preliminary reaction with cellulose, ethanol, and water or directly within the molten PP matrix—yielded composites with significantly lower crystallinity and reduced shrinkage compared to unmodified PP.

The data obtained from rotational rheometry and DSC approved that the hydrolytic polycondensation of ES-40 enhances the processability of the composite melt. This is achieved through the formation of SiO_2_ particles, which disrupt the crystallization process of PP, leading to the crystallinity decreasing of the material. That should be crucial in reducing shrinkage and improving the overall quality of 3D-printed components.

We propose that an optimal composition for these composites involves a carefully calibrated balance of ethyl silicate and cellulose. This ratio is critical for ensuring uniform distribution of ethyl silicate throughout the composite matrix, supported by adequate hydration from the natural moisture content of cellulose to enable effective hydrolytic polycondensation. It is important to recognize, however, that excess cellulose can complicate the processing of the composite, while too much ethyl silicate may result in unreacted additives forming voids, leading to potential defects.

The development of this composite material with reduced crystallinity, enhanced biodegradability, and improved processability underscores its potential for FDM 3D printing applications.

## Figures and Tables

**Table 1:** Prepared sample designations.

Sample	Component Concentration, wt.%			Cellulose-ES-40 Ratio
	Polypropylene	Cellulose	ES-40	
0–0	100	0	0	
100–0	85	15	0	100–0
0–100	85	0	15	0–100
5–95	85	0.8	14.2	5–95
5–95 E	85	0.8	14.2	5–95
10–90	85	1.5	13.5	10–90
10–90 E	85	1.5	13.5	10–90
30–70 E	85	4.5	10.5	30–70
50–50 E	85	7.5	7.5	50–50

## Data Availability

The data that support the findings of this study are available from the corresponding author upon reasonable request.
